# P-1063. Morbidity and Mortality Analysis of Carbapenem-Resistant versus Carbapenem-Susceptible Acinetobacter baumannii infections in patients hospitalized at an Academic Hospital from 2019-2023

**DOI:** 10.1093/ofid/ofaf695.1258

**Published:** 2026-01-11

**Authors:** Alex Belote, Regan Konz, Eric Gregory, Joanna Kimball, Kellie Wark, Gabe Haas

**Affiliations:** University of Kansas Medical Center, Kansas City, Kansas; University of Kansas Medical Center, Kansas City, Kansas; The University of Kansas Health System, Kansas City, MO; The University of Kansas Medical Center, Kansas City, Kansas; University of Kansas, Kansas City, Kansas; Kansas Department of Health & Environment, Topeka, Kansas

## Abstract

**Background:**

Carbapenem-resistant *Acinetobacter baumannii* (CRAB) is an international health concern, leading to prolonged hospital stays and increased mortality. Global data highlight increased financial and clinical impacts of CRAB, reinforcing the need for surveillance and antimicrobial stewardship to combat this growing threat. Data of CRAB infections is sparse within the U.S. regarding mortality, length of stay (LOS), and cost factors.

**Table 1:**
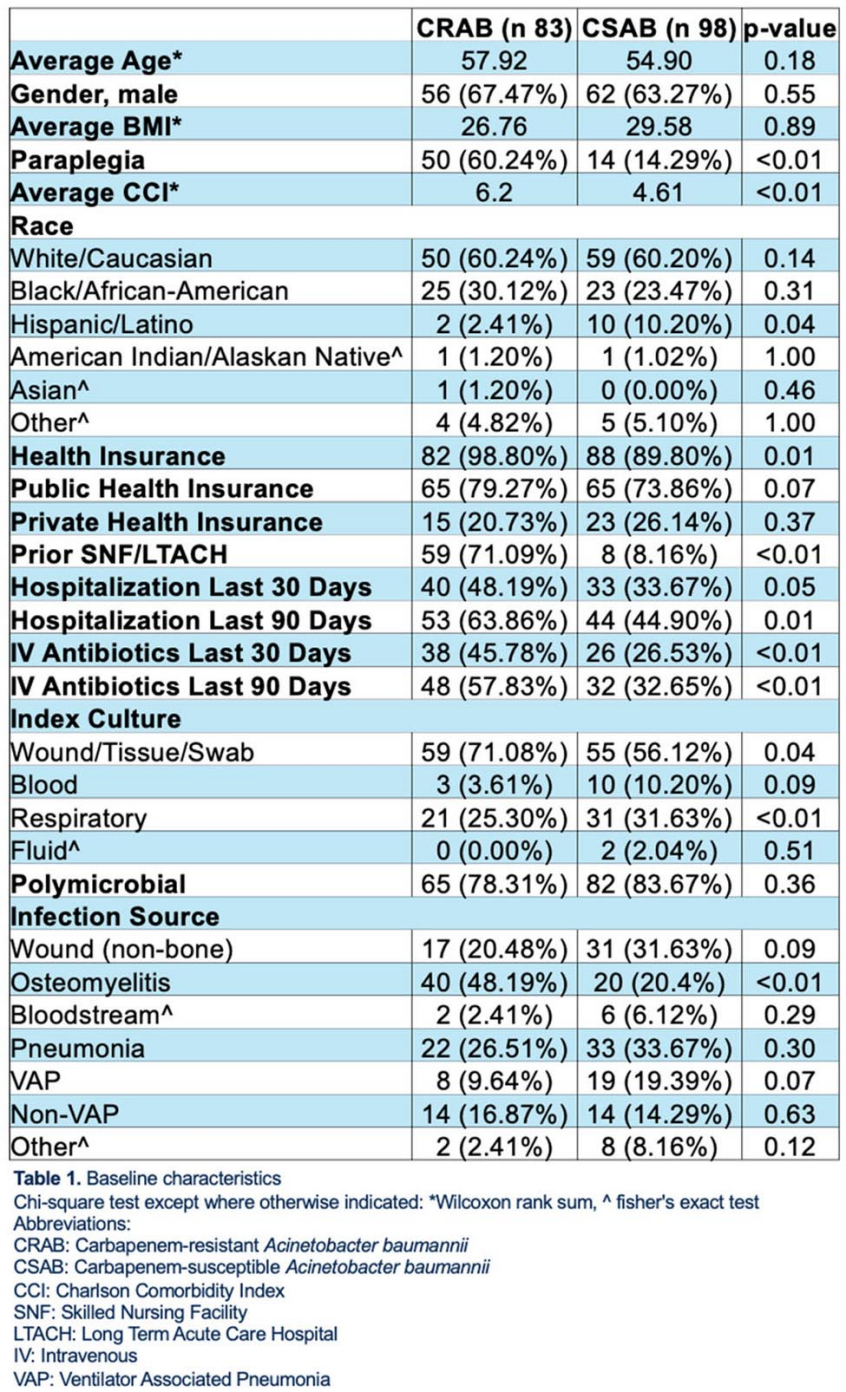
Baseline characteristics

**Table 2:**
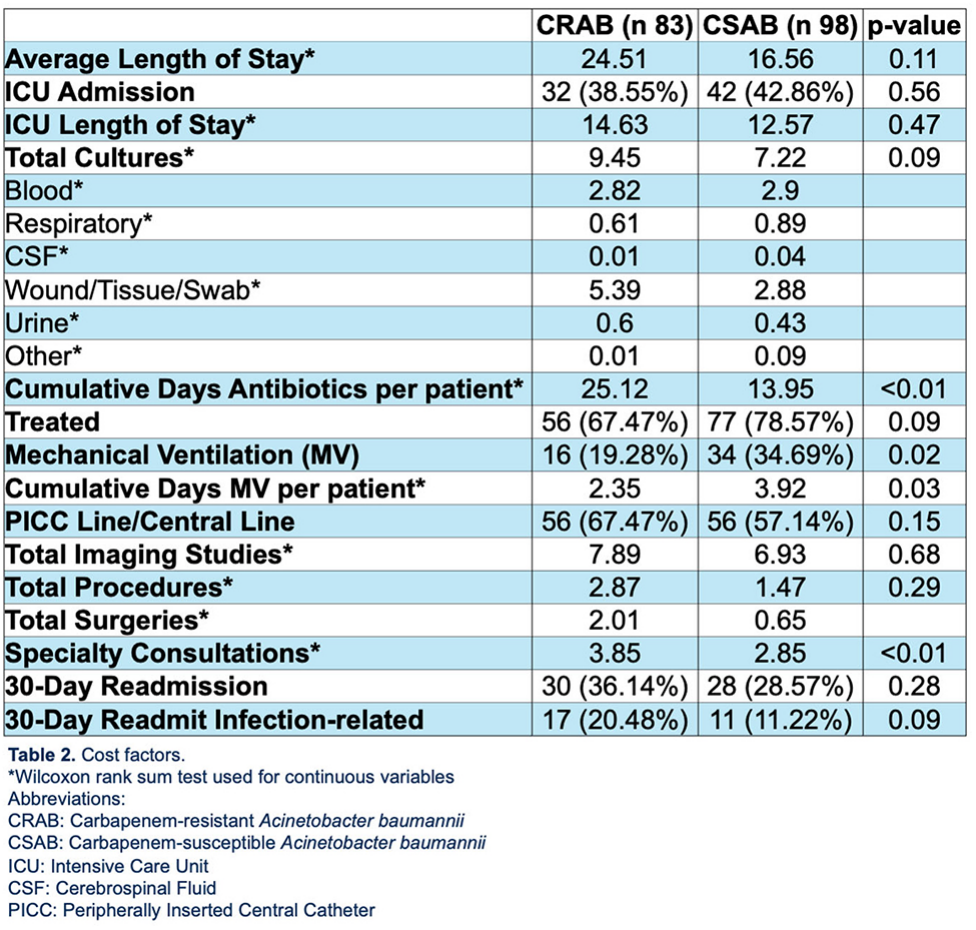
Cost Factors

**Methods:**

All inpatient cases of *A. baumannii* at our institution from 2019-2023 were retrospectively reviewed. Patients aged ≥ 18 with isolation of *A. baumannii* from a non-urinary source with meropenem MIC ≥ 8 or ≤ 2 were included. Primary outcome was 90-day mortality. Secondary outcomes included 30-day mortality, LOS, and additional cost factors. Analysis used JASP Statistical Software. Logistic regression was used to adjust for age, CCI, and infection type.

**Table 3:**
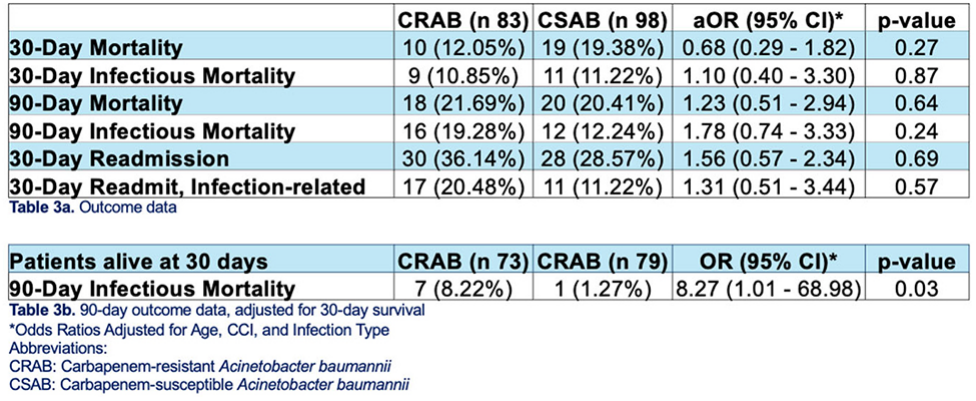
Outcome/Mortality Data

**Table 4:**
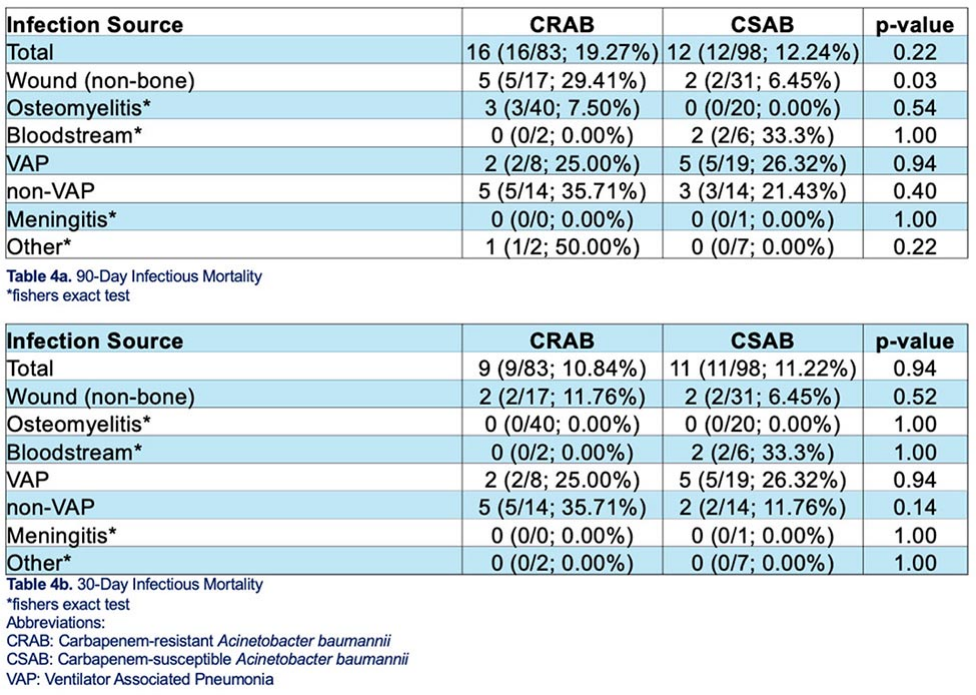
90 and 30-day Infectious Mortality

**Results:**

181 *A. baumannii* isolates were included for analysis. CRAB patients had a higher CCI, rates of paraplegia, recent hospitalizations, recent SNF/LTACH stays, and recent IV antibiotic use, detailed in Table 1. The CRAB cohort had a longer LOS, ICU LOS, longer antibiotic courses (25.12 vs. 13.95 days, p < 0.01), and infectious-related readmissions, detailed in Table 2. CRAB patients had more cultures, imaging, surgeries, and specialty consultations.

Our study indicated no overall significant differences in infectious-related mortality at 90 days (aOR=1.78 ,p=0.240) or 30 days (aOR=1.10, p=0.867) when adjusted for age, CCI, and infection type. However, CRAB had a statistically greater 90-day infectious-related mortality rate if surviving at 30 days (OR=8.27, p=0.022). Additionally, wound infections had significantly greater 90-day infectious mortality for CRAB vs. Carbapenem-susceptible *A. baumannii* (CSAB) (OR=6.04, p=0.03). There were no significant differences in mortality for CRAB vs. CSAB for other infection types.

**Conclusion:**

CRAB infections were linked to greater morbidity and significantly higher resource use. There was no statistically significant difference in 30- and 90-day mortality, though trends indicate higher 90-day infectious mortality in multiple CRAB subgroup analysis. These findings highlight the need for continued *A. baumannii* investigation.

**Disclosures:**

All Authors: No reported disclosures

